# Cryo-EM structure of type 1 IP_3_R channel in a lipid bilayer

**DOI:** 10.1038/s42003-021-02156-4

**Published:** 2021-05-25

**Authors:** Mariah R. Baker, Guizhen Fan, Alexander B. Seryshev, Melina A. Agosto, Matthew L. Baker, Irina I. Serysheva

**Affiliations:** 1 Department of Biochemistry and Molecular Biology, Structural Biology Imaging Center, McGovern Medical School at The University of Texas Health Science Center at Houston, Houston, TX USA; 2 Verna and Marrs McLean Department of Biochemistry and Molecular Biology, Baylor College of Medicine, Houston, TX USA

**Keywords:** Calcium channels, Cryoelectron microscopy

## Abstract

Type 1 inositol 1,4,5-trisphosphate receptor (IP_3_R1) is the predominant Ca^2+^-release channel in neurons. IP_3_R1 mediates Ca^2+^ release from the endoplasmic reticulum into the cytosol and thereby is involved in many physiological processes. Here, we present the cryo-EM structures of full-length rat IP_3_R1 reconstituted in lipid nanodisc and detergent solubilized in the presence of phosphatidylcholine determined in ligand-free, closed states by single-particle electron cryo-microscopy. Notably, both structures exhibit the well-established IP_3_R1 protein fold and reveal a nearly complete representation of lipids with similar locations of ordered lipids bound to the transmembrane domains. The lipid-bound structures show improved features that enabled us to unambiguously build atomic models of IP_3_R1 including two membrane associated helices that were not previously resolved in the TM region. Our findings suggest conserved locations of protein-bound lipids among homotetrameric ion channels that are critical for their structural and functional integrity despite the diversity of structural mechanisms for their gating.

## Abstract (web summary)

3D structure of full-length rat type 1 inositol 1,4,5-trisphosphate receptor reconstituted in lipid nanodisc is determined using single-particle cryo-electron microscopy. The study suggests conserved locations of protein-bound lipids among structurally diverse, homo-tetrameric ion channels.

## Introduction

Inositol 1,4,5-trisphosphate receptors (IP_3_Rs) are tetrameric intracellular cation channels ubiquitously expressed in mammalian cells and located predominantly in the endoplasmic reticulum (ER) membranes. IP_3_Rs are activated by inositol 1,4,5-trisphosphate (IP_3_), a second messenger produced through hydrolysis of phosphatidylinositol 4,5-bisphosphate (PIP_2_) by phospholipase C, which is activated in response to diverse cellular stimuli such as hormones, growth factors, neurotransmitters, neurotrophins, odorants, light, etc.^1–3^. The resulting Ca^2+^ release from the ER via IP_3_R channels elevates the cytoplasmic free Ca^2+^ concentration, a signal that triggers markedly diverse cellular actions, ranging from contraction to secretion, from proliferation to cell death. The most notable feature of IP_3_R channels is biphasic regulation^1,2^ by Ca^2+^, such that at concentrations in the nanomolar range (resting levels in cellular cytosol) Ca^2+^ stimulates IP_3_R-mediated Ca^2+^ release from intracellular stores. In contrast, at higher micromolar concentrations of Ca^2+^ IP_3_R-mediated Ca^2+^ release is inhibited by Ca^2+^. To understand how IP_3_R channels convey their gating through the interplay of its two primary agonists, IP_3_ and Ca^2+^, structures of IP_3_Rs in both the apo- and ligand-bound states have been determined at near-atomic resolutions by single-particle electron cryo-microscopy (cryo-EM). These structures were determined in a detergent-based aqueous environment, allowing for channel solubility^4–7^. However, ion channels reside in biological membranes, and lipids are proven to play important physiological roles in maintaining their structural-functional integrity. Although the latter structures described the IP_3_R architecture and the structural determinants for its function, the structural basis for how IP_3_Rs function in a physiological lipid membrane environment and how lipids affect intrinsic channel protein tasks, such as ligand-binding and gating, remain unknown.

In this study, we present an atomic model of the full-length rat IP_3_R1 channel that was built based on the 3.30 Å resolution cryo-EM map of the channel embedded in a lipid nanodisc. In addition, the structure of IP_3_R1 solubilized with detergent in the presence of phospholipids was solved to 2.96 Å resolution also using the single-particle cryo-EM approach. Structural comparisons of the channels visualized in these two different milieus in a ligand-free state provide insights into IP_3_R1-lipid interactions enabling a deeper understanding of IP_3_R channel function in a physiologically relevant membrane environment.

## Results

### Overall structure of IP_3_R1 in a lipid bilayer

To study the structure in a lipid bilayer environment, we first optimized the purification of full-length IP_3_R1 from rat cerebellum utilizing our previously described procedure that produces functional channels, as demonstrated by IP_3_-induced Ca^2+^ fluxes after reconstitution into lipid vesicles^8^. In this study, the cerebellar microsomal membranes were solubilized with Lauryl Maltose Neopentyl Glycol (LMNG) in the presence of phospholipids (see “Methods” section). We next reconstituted LMNG-solubilized IP_3_R1 into lipid nanodiscs composed of MSP1E3D1/POPC, which form nanodiscs of ~12–14 nm diameter^9^ and are sufficient to accommodate the IP_3_R1 transmembrane (TM) domains without imposing spatial constraints. Importantly, embedding IP_3_R1 into nanodiscs and protein purification was essentially a one-step procedure using immunoaffinity chromatography (Supplementary Fig. 1a; see “Methods” section), minimizing exposure of the solubilized channel protein to detergent and avoiding further displacement of lipid molecules while easily reconstituting the purified channel particles in a near-native lipid environment.

Both IP_3_R1 reconstituted in lipid nanodiscs (IP_3_R1-ND) and IP_3_R1 solubilized in LMNG (IP_3_R1-LMNG) preparations were analyzed by cryo-EM under similar conditions in the presence of EGTA and without the addition of any channel-specific ligands. Corresponding cryo-EM images showed monodisperse samples with particles evenly distributed across the hole (Supplementary Figs. 1b and 2a). Side views of the channel exhibit a characteristic “screw-shape” displaying the TM and cytoplasmic (CY) regions and are easily recognizable. 2D class-averages generated from both samples illustrated different particle orientations with many features in both the CY and TM domains (TMDs) (Supplementary Figs. 1c and 2b). 3D reconstructions were calculated for both data sets at an average nominal resolution of 3.30 Å for IP_3_R1-ND and 2.96 Å for IP_3_R1-LMNG using the “gold standard” Fourier shell correlation (FSC) = 0.143 criterion (Supplementary Figs. 1f, 2e; Table 1; see “Methods” section). However, the local resolution in most of the TM domains and some of the CY domains reached to better than 2.8 Å resolution that enabled reliable assignment of the side chains in these regions (Supplementary Figs. 1g and 2f).

**Table 1:** Cryo-EM structure determination and model statistics.

	IP_3_R1-ND	IP_3_R1-LMNG
	EMDB-23337	EMDB-23338
	PDB-7LHE	PDB-7LHF
Data collection		
Microscope	Titan Krios G3	Titan Krios G3
Voltage (kV)	300	300
Detector	Gatan K2 Summit	Gatan K2 Summit
GIF energy filter (eV)	20	20
Magnification	130,000	130,000
Pixel size (Å)	1.07 (0.535)	1.07 (0.535)
Total dose (e/Å^2^)	56	42
Dose rate (electrons/Å^2^/s)	8	6
Exposure time (sec)	7	7
Defocus range (μm)	−0.8 to −3.5	−0.8 to −3.5
Movie stacks	22,000	19,105
Subframes	35	35
Data processing		
Defocus determining software	CTFFIND3	GCTF
Motion correction software	Motioncorr2	Motioncorr2
Refine software	cryoSPARC	RELION 3.0
Particle picking script	Template picker	e2boxer.py
Number of boxed particles	4,801,610	1,407,714
Number of particles after 2D classification	1,495,402	1,011,190
Number of particles in final reconstruction	573,723	303,481
Symmetry imposed	C4	C4
Map resolution (Å)	3.3	2.96
Atomic model		
Ramachandran outliers (%)	0.25	0.08
Ramachandran favored (%)	88.91	89.65
Rotamer outliers (%)	0	0.05
C-beta deviations	0	4
RMS (bonds)	0.0044	0.0045
RMS (angles)	1.04	1.05
Molprobity score	1.92	1.91
Molprobity clashscore	6.21	6.36

**Fig. 1:**
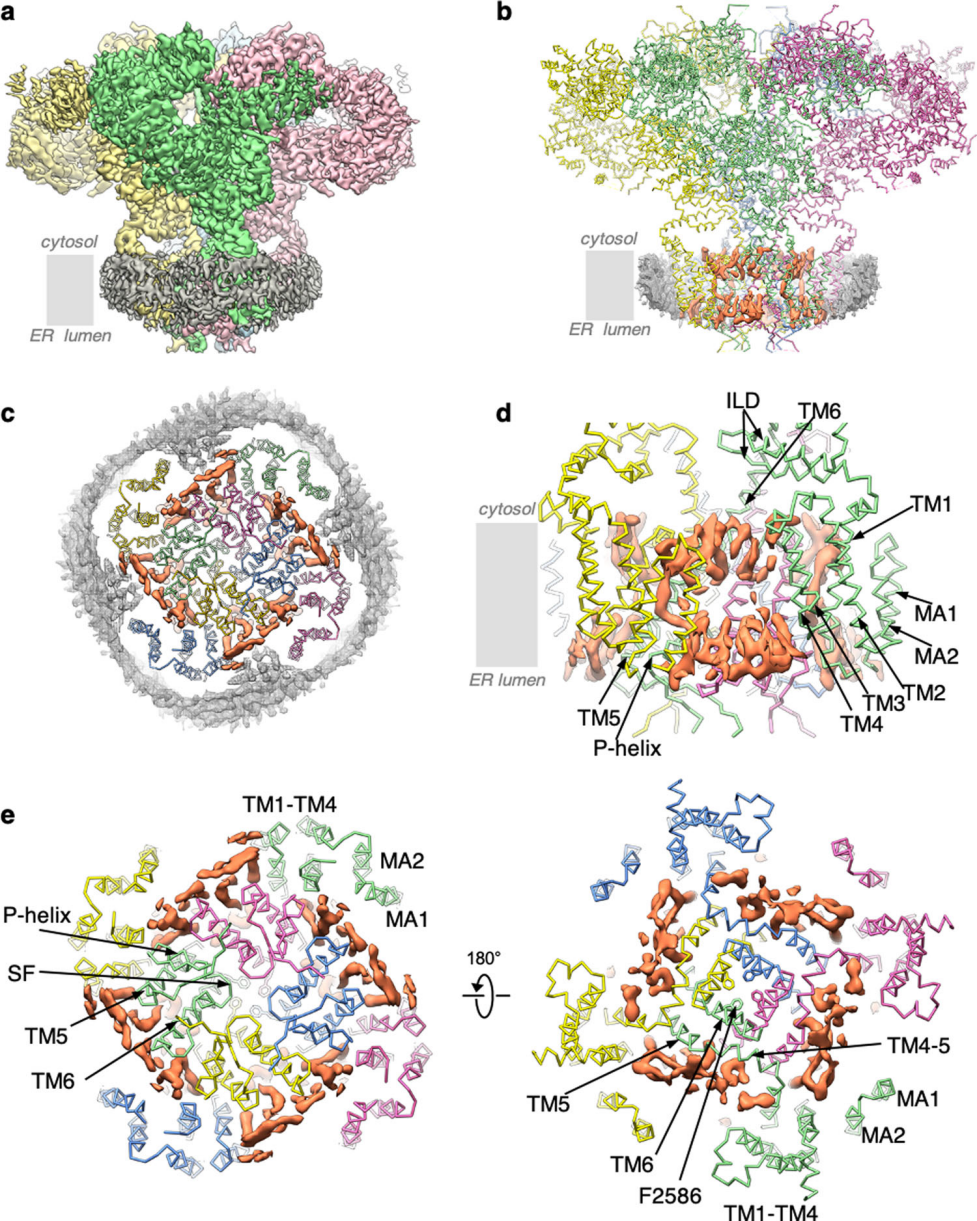
Overview of lipids in the IP_3_R1-ND structure. **a** The cryo-EM density map of the IP_3_R1-ND complex is viewed along the membrane plane. Subunits are color-coded and lipid-nanodisc densities are colored gray. **b** The atomic model of IP_3_R1-ND viewed along the membrane plane. A slice through the nanodisc densities (gray mesh) reveals the protein-bound lipid densities (orange). The model of IP_3_R1 is shown in wire representation and color-coded by subunit. **c** TM region of IP_3_R1 in nanodiscs viewed from the lumen along the channel’s four-fold symmetry axis. **d** TM region viewed along the membrane plane; the lipid densities are shown in orange and displayed at 5σ threshold. **e** Zoomed-in view of lipid densities in inter- and intra-subunit crevices viewed along the four-fold axis from the lumen (left) and cytosol (right). The side-chain of F2586 that constitutes the gate in TM6 is displayed.

The overall architecture of both IP_3_R1-ND and IP_3_R1-LMNG are nearly identical to each other and to the previously determined cryo-EM structures of the IP_3_R1 channel in a ligand-free, closed state (Fig. 1a and Supplementary Figs. 5a, 6a–c, Supplementary Movie 1)^4,5^. The IP_3_R1 protein comprises a large CY region with nine well-defined domains: two β-trefoil domains (β-TF1 and β-TF2), three armadillo solenoid folds (ARM1-ARM3), a helical domain (HD), an intervening lateral domain (ILD), and a linker domain (LNK), that form a solenoid scaffold around the central four-helix bundle made of the cytosolic C-terminal domains (CTDs) from each subunit in the tetrameric channel. The transmembrane pore-forming region exhibits a domain-swapped architecture (Fig. 1c, e), in which the peripheral TM1–TM4 helical bundle of one subunit interacts with the central pore-lining TM5 and TM6 helices of a neighboring subunit. This domain-swapped arrangement is likely made possible by the relatively long lateral TM4-5 helix. Notably, the cryo-EM density maps of IP_3_R1 solved in this study are of higher quality than any of the IP_3_R structures reported to date^4–7^. The improved density features were observed throughout the entire channel protein, and most of the protein domains exhibit well-resolved side-chain densities (Supplementary Figs. 3 and 4) to allow for unambiguous building of atomic models for ~83% of the protein, including additional structural elements in the TM region that were not previously resolved (see “Methods” section; Supplementary Table 1)^4–7^. Both IP_3_R1-ND and IP_3_R1-LMNG structures showed the additional protein densities at the lumenal vestibule that were discerned as two antiparallel, membrane-associated (MA) helices, MA1 and MA2, that form a membrane-embedded helix-loop-helix structure comprising residues P2307–G2349 (Fig. 3b and Supplementary Movie 2). The MA2 helix was partially resolved in our previous study^5^. It is conceivable that the presence of lipids stabilizes structural elements of the TM region enabling their unambiguous determination. The MA1–MA2 structure is positioned ~14 Å from the center of the TM1–TM4 helical bundle and tilted ~24° away from it (Supplementary Fig. 6d, e). Notably, in the IP_3_R1-LMNG structure, the MA1 helix proximate to the lumenal side of the membrane has a 14 Å displacement from MA1 in IP_3_R1-ND.

The cryo-EM map of IP_3_R1-ND clearly shows densities consistent with the presence of a lipid nanodisc that forms a round-shaped matrix surrounding the entire TM region and delineates lipid bilayer boundaries with well-defined cytosolic and lumenal leaflets (Fig. 1a–c). The lipid nanodisc measures about 30 Å high × 130 Å wide, which is in agreement with previously reported nanodisc dimensions^9^. We observed several “hairpin-shaped” and elongated densities, tightly adhering to the TM domains and most often appearing perpendicular to the membrane plane (Fig. 1b–e and Supplementary Movie 1). Notably, these well-ordered densities were observed in both the IP_3_R1-ND and IP_3_R1-LMNG maps (Supplementary Fig. 5b, c). The shape of these densities is consistent with phospholipid molecules. These lipid-like densities were putatively modeled as phosphatidylcholine (PC), the major component (>50%) of ER membranes^10^, which was added during protein solubilization and nanodisc formation (see “Methods” section).

### Ion-permeation pathway in IP_3_R1–lipid complexes

**Fig. 2:**
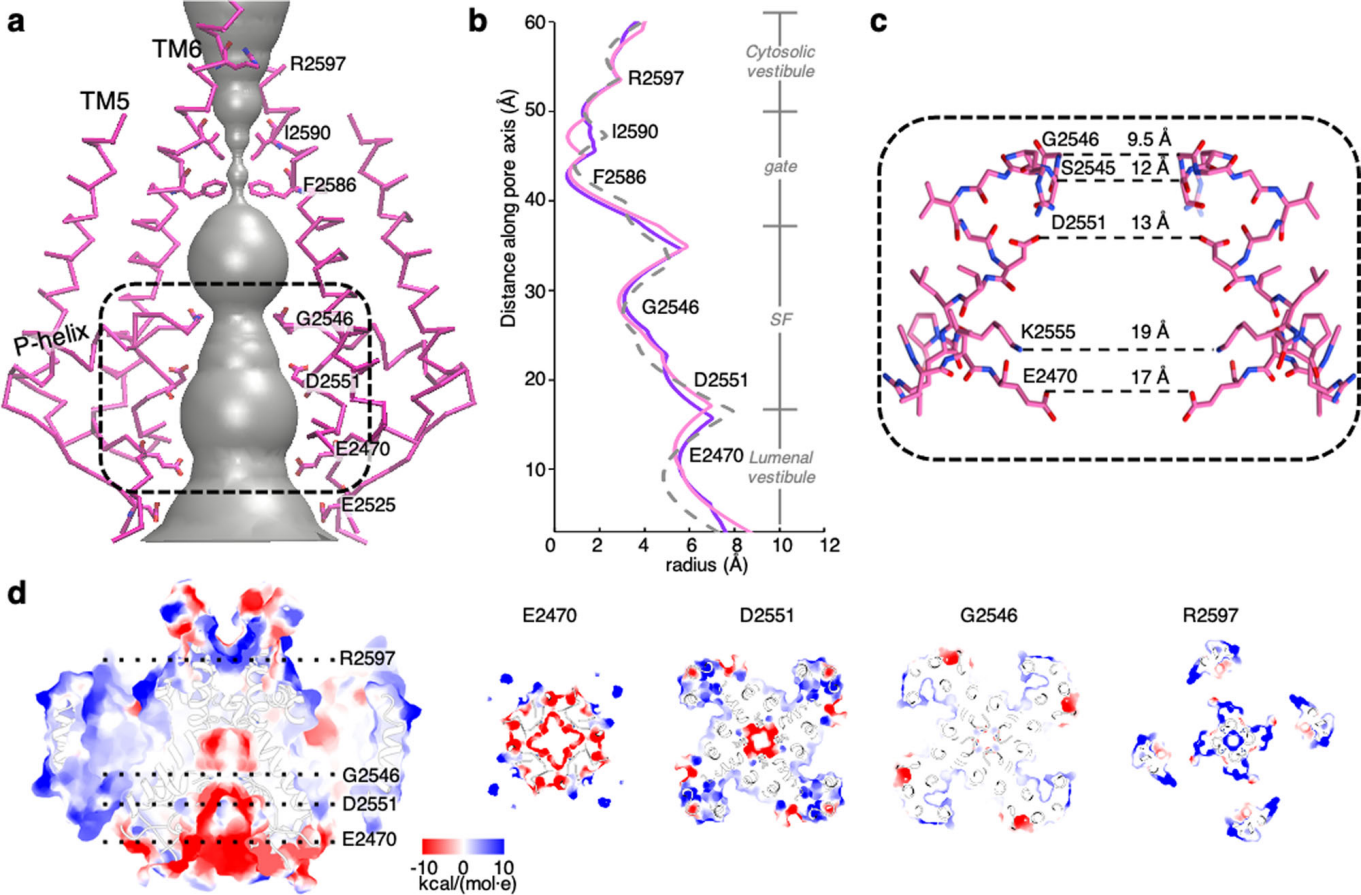
The ion-permeation pathway of IP_3_R1 in nanodisc. **a** Solvent-accessible pathway along the IP_3_R1-ND pore mapped using the program HOLE^54^. A series of residues within the ion-conduction pathway are labeled. Dashed line box indicates the zoomed-in region in **c**. **b** Comparison of the pore dimensions for IP_3_R1-ND (pink), IP_3_R1-LMNG (purple), and IP_3_R1-CHAPS (PDB ID: 6MU2; dashed gray line). **c** A wire representation of the SF in IP_3_R1-ND; two opposing subunits are viewed along the membrane plane; narrowest distances between Cα atoms (G2546) or side-chain atoms along the SF are indicated. **d** The surface electrostatic potential along the ion-permeation pathway. The left panel shows a slice through the channel pore along the four-fold axis with the location of slices perpendicular to the four-fold axis (right panels) indicated by dotted lines. Panels on the right show slices through the pore at E2470, D2551, G2546, and R2597 viewed from the cytosolic side along the symmetry axis.

**Fig. 3:**
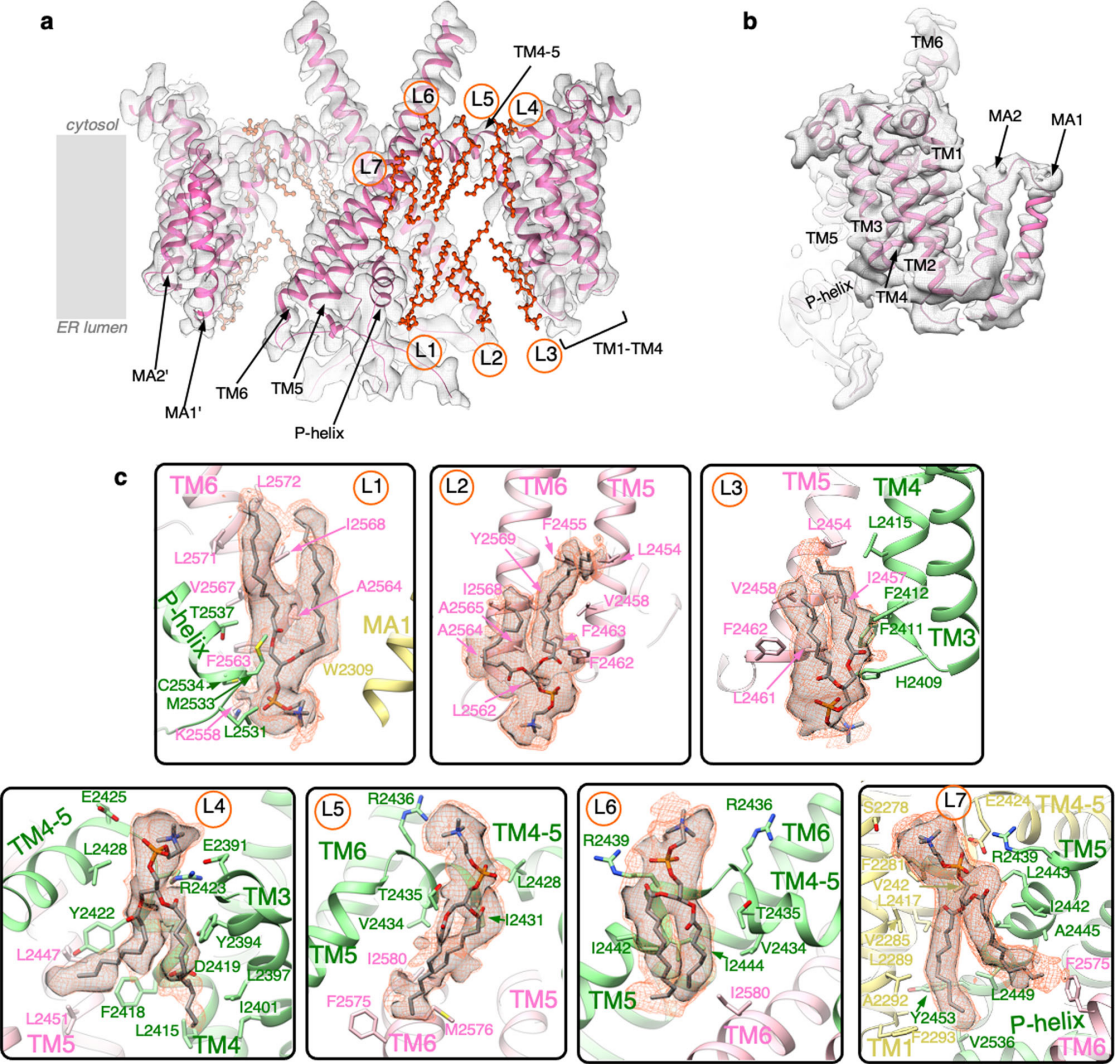
Protein–lipid interactions identified in IP_3_R1-ND structure. **a** IP_3_R1-ND cryo-EM density map for TM1–TM6 helices of two opposing subunits; the model is depicted as a ribbon and viewed parallel to the membrane plane. Lipids are represented as orange ball-and-stick models and labeled L1–L7. **b** TMD densities for one subunit are overlaid with the IP_3_R1-ND model viewed parallel to the membrane plane and rotated ~90° from the position in **a**. **c** Zoomed-in views of seven lipid-binding sites (L1–L7) identified in the TM region. Lipids are putatively modeled as phosphatidylcholine (see “Methods” section); the PC models are colored by elements and fit into corresponding cryo-EM densities displayed at 3–5σ cut-off values (gray) and 2–2.5σ (orange mesh). TM helices are depicted as ribbons colored by channel subunit. Residues within 5 Å of the lipid molecules are displayed in a stick representation and labeled.

Consistent with the previous studies, the ion-permeation pathway in IP_3_R1 is formed around the central axis of the tetrameric channel assembly and defined by the pore-lining TM5 and TM6 helices, the short reentrant pore helix (P-helix), and pore loop containing the selectivity filter (SF) (Fig. 2a, c)^4,5^. The calculated solvent-accessible ion-conduction profile shows that the residues F2586 and I2590 form two constrictions with the distances of 1.4 and 1.4 Å in IP_3_R-ND and 1.4 and 3.6 Å in IP_3_R-LMNG, respectively (Fig. 2a, b). This pore conformation is remarkably similar to the pore architecture in the previous structures of apo-IP_3_R1 and is designated as a closed pore conformation^4,5^. Notably, seven lipid molecules per subunit were revealed in the TM region of IP_3_R1, regardless of whether the channel was reconstituted in lipid nanodiscs or solubilized in LMNG (Fig. 3a, c and Supplementary Figs. 5b, c, 6d, e). Moreover, the structural comparisons of the density maps and models of IP_3_R1-ND and IP_3_R1-LMNG revealed that lipid molecules are tightly associated with the TM domains at nearly identical locations in both structures. The locations of ordered lipid densities mimic the lipid bilayer and form extensive interfaces with the TMDs appearing in inter- and intra-subunit crevices. The lipid buried surface area accounts for 10,307 Å^2^ and 9,866 Å^2^ in the TMDs of IP_3_R1-ND and IP_3_R1-LMNG, respectively. The lipid–protein interfaces exhibit hydrophobic packing that appears to maintain a seal across the membrane. Due to the domain-swapped architecture of the TMDs, some lipid-binding sites involve multiple IP_3_R1 subunits.

Lipid densities (L1–L3) were observed in the hydrophobic crevices formed close to the lumenal vestibule of the ion-permeation pathway of IP_3_R1 (Fig. 3a, c). One lipid molecule (L1) is found at a junction between three IP_3_R1 subunits: the central pore-lining TM6 and P-helix of two neighboring subunits and the MA helices from another subunit (Fig. 3c). The intra-subunit binding site for L2 is formed by the hydrophobic and aromatic residues of TM5 and TM6 helices with one acyl chain contacting TM6 and the other acyl chain intercalating between the lumenal ends of TM5 and TM6 (Fig. 3c). L3 fills the lumenal crevice between the TM4 and TM5 helices of two neighboring subunits. H2409 is in a position to potentially form a salt bridge with the L3 headgroup (Fig. 3c).

It appears that the lipid environment stabilizes the lumenal vestibule of the channel, so that the P-helix and SF, defined as ^2546^GGGVGD^2551^, are well resolved in both the IP_3_R1-ND and IP_3_R1-LMNG structures (Fig. 2a, c and Supplementary Fig. 7a–c). In the apo-state, Ca^2+^ ions from the ER lumen can access the channel’s lumenal vestibule and encounter negatively charged residues (E2525, E2470) lining the entrance to the ion-conduction pathway. These residues contribute to an electronegative environment that may hold or stabilize cations in the aqueous vestibule before they traverse across the lipid bilayer (Fig. 2d). In the non-conducting (apo-) state, the SF is defined by the side chains of D2551 and S2545 along with the Cα of G2546 constituting the narrowest point of the SF (Fig. 2c). The distance across the SF is ~13 Å at D2551 and ~9.5 Å at G2546. This implies that the selectivity filter in both the IP_3_R1-ND and IP_3_R1-LMNG does not constitute a barrier that would preclude the passage of fully hydrated Ca^2+^ ions^11^. In previous work, by removing the carboxylic acid group of the aspartic acid residue, the D2551A mutation creates a pore dead channel unable to translocate Ca^2+^ ions, yet the conservative mutation, D2551E, alters the channel selectivity for divalent cations to favor monovalent ions^12^. This suggests that the D2551 side chains from each subunit could form a single Ca^2+^ binding site that might be responsible for the weak selectivity of IP_3_R1 for Ca^2+^ over Na^+^ or K^+^ (Fig. 2c). Moreover, a mutation in the SF of the corresponding glycine residue to arginine of human IP_3_R1 results in Spinocerebellar Ataxia Type 29 and Gillespie Syndrome (Fig. 5)^13^ and in vitro G2546A mutations eliminate Ca^2+^ flux^14^. Interestingly, the highly conserved, positively charged R2544 is positioned at the C-terminal end of the P-helix, which would not seem conducive to the rapid movement of cations through the channel. However, the side-chain of R2544 points towards the TM6 helix of the neighboring subunit and is positioned to form a salt bridge with D2570 (Supplementary Fig. 7d). It was found that mutating this charged residue to alanine alters Ca^2+^ regulation of the receptor^14^. Specifically, R2544A channels appeared to be activated normally by low concentrations of Ca^2+^, but exhibit enhanced inhibition by high Ca^2+^ when compared with wild-type channels. However, the R2544A mutation has little effect in ^45^Ca^2+^ flux assays^12^, suggesting that the R2544 side-chain projects away from the permeation path, which is in good agreement with our IP_3_R1 structures. Altogether, these studies suggest that mutations within the P-helix resulting in structural changes in the SF might regulate ion flow through IP_3_R channels. A similar regulatory mechanism by conformational changes in SF has been observed in K^+^ channels^15,16^.

Three lipid molecules (L4–L6) are found lining the amphipathic TM4-5 helix that runs parallel to the membrane plane at the cytosolic leaflet of the lipid bilayer and connects the peripheral bundle of the TM1–TM4 helices with the central pore-lining TM bundle. The phospho-head groups of the lipids are located at the boundary of lipid bilayers, in a position to potentially interact with polar residues from the channel. L4 occupies an intra-subunit hydrophobic cleft formed at the interface between the TM3, TM4, and TM4-5 helices (Fig. 3c). Both hydrocarbon chains of L4 appear to nestle among the hydrophobic side chains of TM3 and TM4 while the bulkier density of the polar head group is located next to the N-terminus of the TM4-5 horizontal helix at the lipid/water interface. Mutations of charged residues in the TM4-5 helix (D2419A, R2423A, E2424A, E2425A) have been found to inhibit channel function, pointing to the importance of this domain for the channel activation^17^. Proper lipid integration into the TM4-5 helix may play a critical mechanistic role in the channel gating. Structures of several other tetrameric cation channels (such as TRPV1, TRPV3, NOMPC, and K_V_1.2), have also shown lipid binding at this junction and this lipid plays a regulatory role in K_v_1.2 channel function (Supplementary Fig. 8)^18^. The polar head group of another lipid (L5) is positioned near R2436, while its two hydrocarbon tails curve under the TM4-5 helix and continue toward the pore-lining TM5 and TM6 helices of the neighboring subunit (Fig. 3c). Noteworthy, the TM6 helices in the lipid-bound structures exhibit a conformation satisfying a canonical α-helix hydrogen bonding, however, the hydrocarbon tail of L5 is observed near the site where a π-helix bulge in TM6 (residues M2576, I2580) was observed under activating conditions^5^. L6 is located between TM4-5 and TM5 helices with the phospho-head group residing between two arginine residues, R2436 and R2439, which could provide favorable electrostatic interactions between the negatively charged lipid headgroup and the channel protein (Fig. 3c). The two branches of the hydrocarbon tail traverse ~2 turns down the TM5 helix to I2442, filling in a hydrophobic pocket formed by TM5 with TM6 of the neighboring subunit.

A long, ovoid density attributed to L7 is found at the inter-subunit interface between the TM5 helix and TM1 and TM4 helices from the neighboring subunit (Fig. 3c). Both hydrocarbon tails of L7 are well resolved with one branch of the acyl chain occupying the majority of the hydrophobic pocket formed by TM5, TM1, and TM4. The second hydrocarbon chain fills a hydrophobic pocket between the TM5 and TM6 helices of the neighboring subunits. It is noticeable that the well-defined bulky phospho-head group of L7 runs ~6 Å deeper into the cytosolic leaflet of the lipid bilayer than L4–L6. This lipid placement may better execute a stabilizing role on TMDs supporting optimal channel gating.

### Allosteric nexus at cytosolic–lipid bilayer interface

**Fig. 4:**
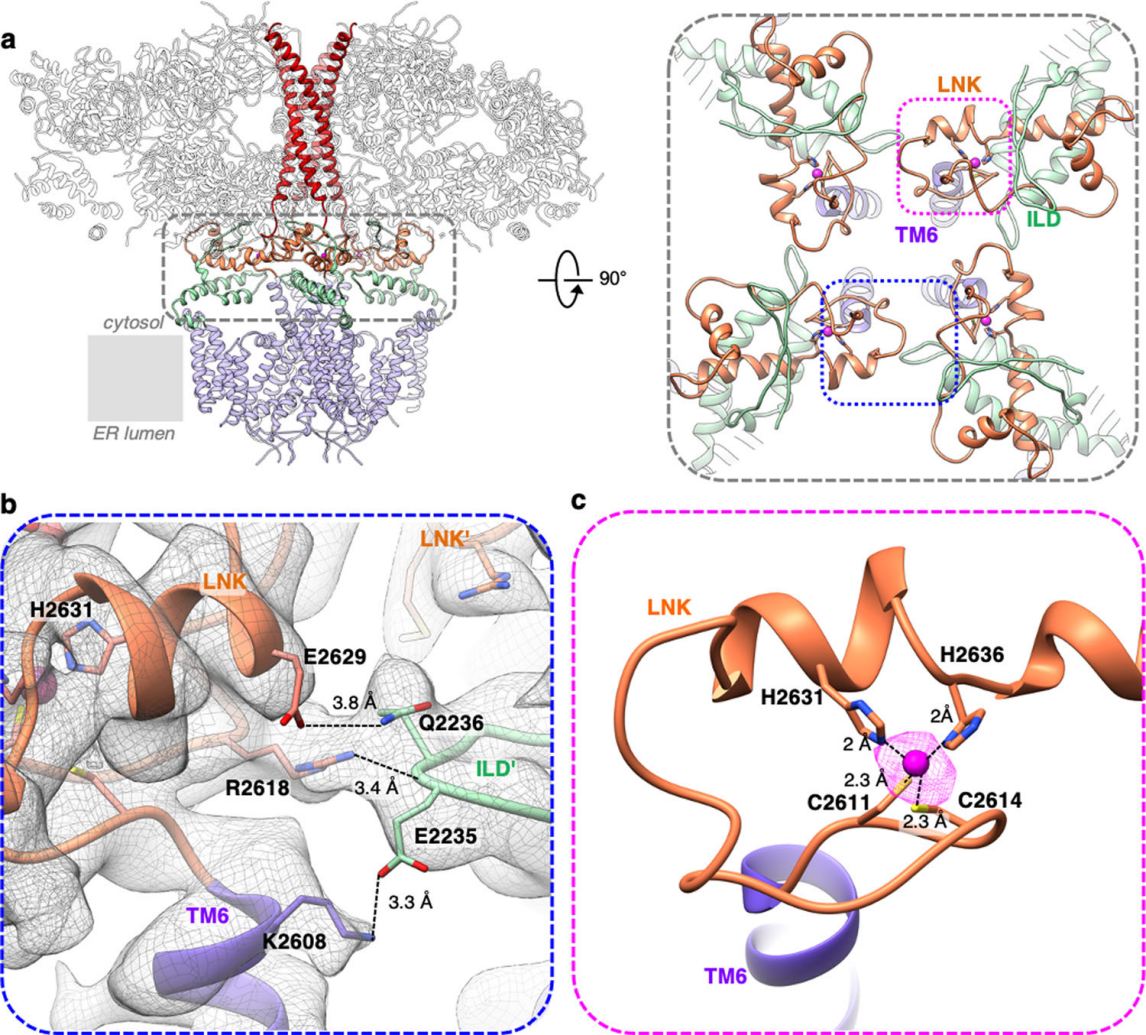
IP_3_R1 domains at the cytosolic-lipid bilayer interface. **a** The ILD (light-green) and LNK domain (orange) form a metastable nexus connecting the CY (gray) and TM (purple) regions in the tetrameric IP_3_R1 channel; viewed along the membrane plane. The right panel shows a zoomed-in view of the domains (indicated by the gray dashed line in the left panel) at the cytosolic-membrane interface viewed from cytosol along the four-fold axis. **b** The interface between ILD and the LNK/TM6 of neighboring subunits overlapped with corresponding EM densities (displayed at 4σ). **c** Close-up view of Zn^2+^ binding site in LNK domain; residues in the C2H2-like Zn^2+^ finger are labeled; the density corresponding to Zn^2+^ is depicted as magenta mesh, contoured at 15σ.

Improved resolvability in the density maps was not limited to TM regions, but also extended to the CY regulatory domains. The intervening lateral (ILD) and linker (LNK) domains form a metastable nexus above the channel’s cytosolic vestibule that represents the only link between the CY and TM domains and is essential for the channel gating (Fig. 4a)^4,5^. Our previous studies demonstrated that the ILD/LNK assembly undergoes substantial structural rearrangements upon IP_3_R1 activation and is responsible for the transmission of ligand-evoked signals to the pore^5^. Although the ILD/LNK structure of the apo-IP_3_R1 in lipid bilayer is similar to that in detergent^4–7^, it provides an immense amount of structural details, including unambiguous side-chain placement, revealing putative hydrogen bonding within the domains and coordination of a Zn^2+^ ion in its binding pocket^4^.

**Fig. 5:**
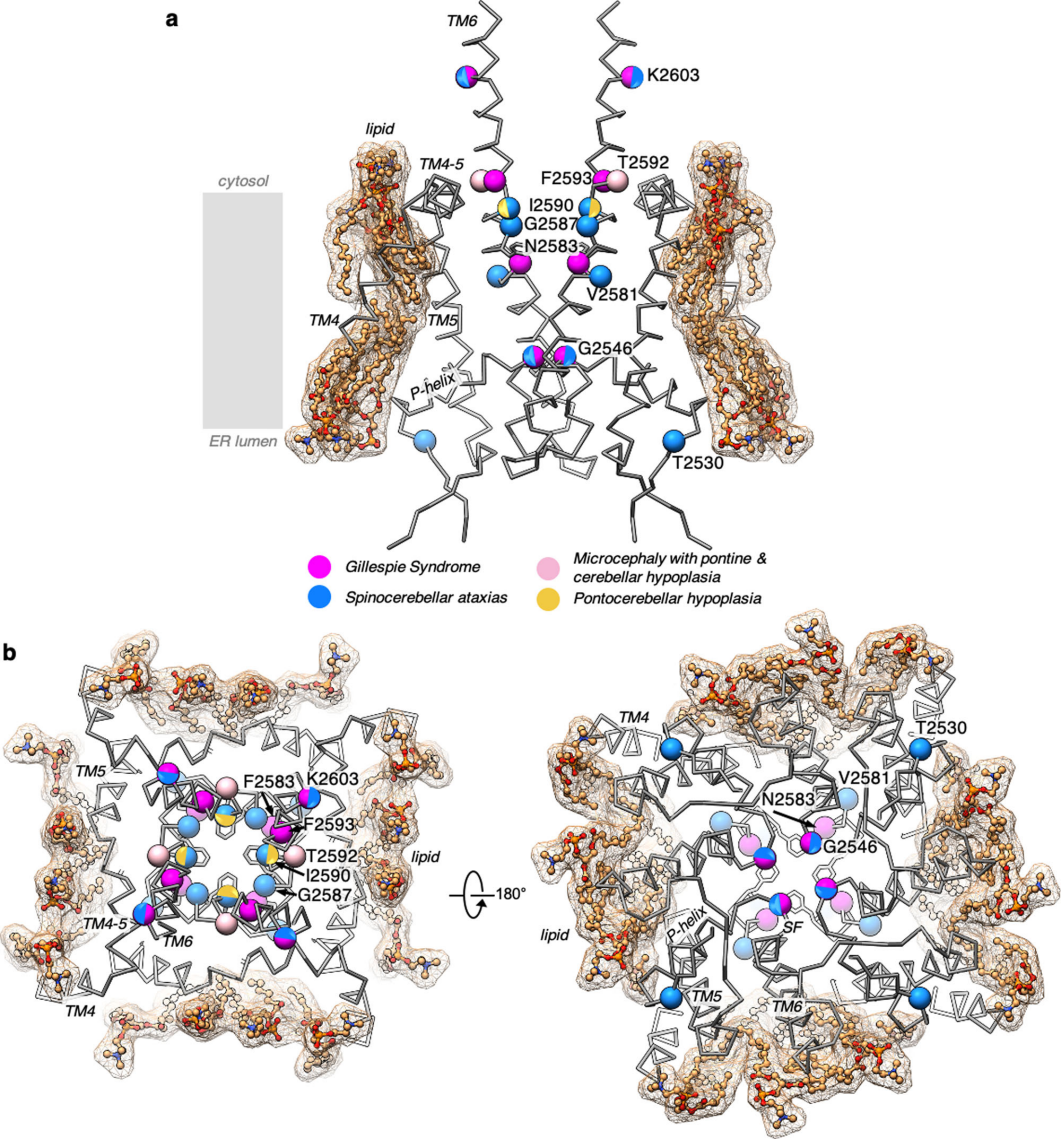
Model of disease-associated mutations in TM domains of IP_3_R1. **a** TM4–TM6 helices (gray, wire representation) from two opposing subunits of IP_3_R1-ND are shown in side view; lipid molecules (orange) are depicted as ball-and-stick models overlapped with corresponding surface representations (mesh). Residues in the rat IP_3_R1 sequence that correspond to the human IP_3_R1 mutations associated with Gillespie Syndrome, microcephaly with pontine and cerebellar hypoplasia, spinocerebellar ataxias, and pontocerebellar hypoplasia are depicted as spheres and colored magenta, pink, blue, and yellow, respectively. **b** TM4–TM6 helices of four subunits with lipids viewed from the cytosol (left panel) and lumen (right panel) perpendicular to the membrane plane.

A unique feature in the superfamily of intracellular Ca^2+^ release channels, which includes the closely related IP_3_Rs and RyRs, is the intra-subunit layering of two non-contiguous domains with the C-terminal LNK domain sandwiched between two β-strands and the helix-turn-helix motif of the ILD domain, which connects the ARM3 and TM1 domains (Fig. 4a and Supplementary Fig. 6a). The ILD/LNK domains are positioned to couple multiple cytosolic regulatory signals to gating motions of the channel^4,5^. Based on our structural analysis of IP_3_R1 in a lipid environment, potential non-covalent interactions can be proposed within the ILD/LNK nexus that might be essential for stabilization of ILD/LNK assembly providing structure-functional integrity of the channel (Fig. 4b)^19^. Specifically, R2618 and E2629 from the LNK domain are in a position to form hydrogen bonds with E2235 and Q2236, respectively, which reside on the ILD of the neighboring subunit. Additionally, K2608 from the cytosolic extension of TM6 is in a position to form a salt bridge with E2235, further linking the ILD/LNK domains with the TMD. The deletion of the nearby residue K2603 (K2563 in human IP_3_R1) at the cytosolic extension of TM6 has been linked to Gillespie Syndrome and causes dysfunctional IP_3_R1 channels that are unable to release Ca^2+^ (Fig. 5)^20,21^. This deletion would effectively shorten the length of TM6 likely altering the intermolecular contacts of the ILD/LNK nexus with the TMDs.

Four Zn^2+^-binding sites in the tetrameric channel are defined by a C2H2-like Zn^2+^ finger motif (C2611, C2614, H2631, and H2636) in each IP_3_R1 subunit. The cryo-EM maps determined in a lipid environment show strong density peaks (>15 σ) within the binding pocket, which we have assigned as Zn^2+^ ions (Fig. 4c and Supplementary Fig. 9). The Zn^2+^-binding site shows a classic tetrahedral coordination revealing the mechanism of stabilization due to Zn^2+^ binding. Zn^2+^ binding at the C2H2-like motif appears to be integral to the native IP_3_R1 structure, as purification of the channel was conducted in the presence of cation chelators and no additional Zn^2+^ was added. Of note, the stability of the tetrameric structure of IP_3_R is directly tied to sequences within the channel’s C terminus, which includes the C2H2-like Zn^2+^ finger in the LNK domain^22^.

## Discussion

In the present study, we have solved the first structure of the full-length neuronal IP_3_R1 channel reconstituted in lipid nanodiscs, which provides a native-like lipid bilayer environment suitable for cryo-EM structural characterization of the membrane protein in a soluble, detergent-free form. We have also determined the structure of IP_3_R1 solubilized in LMNG in the presence of phospholipid in order to assess to what extent the detergent-solubilized channel adopts a native structure when compared with the channel embedded in a membrane environment. Our work was motivated by the release of numerous cryo-EM structures of many tetrameric ion channels obtained both in the aqueous detergent-based and lipid-based environments^23^. Based on these studies, it has become evident that the environmental conditions, specifically the lipid bilayer, are responsible for many of the structural differences identified, *e.g.*, the TM pore-lining S6 helix of the TRPV3 channel^24–27^ and the VSLD and peripheral TM helices of the RyR1 channel^28^.

For IP_3_R1, we found that our two structures, with the channel embedded in lipid nanodiscs or solubilized in LMNG in the presence of phospholipid, are virtually identical with only subtle structural differences identified in the CY and TM domains, as well as a displacement of the peripheral membrane-associated helices (Fig. 3a, b and Supplementary Figs. 6b–e, 7a–c). These differences can be accounted for by the embedding medium in cryospecimens (detergent-free *vs.* detergent-solubilized) and may conceivably be due to the lack of the full lipid shell in IP_3_R1-LMNG structure. Strikingly, both cryo-EM structures of IP_3_R1 presented here revealed well-ordered lipid densities that are immersed at near-identical locations in cavities and clefts formed by the TM domains. However, the IP_3_R1-ND structure likely represents a more complete picture of the lipid topology in IP_3_R1 that includes the nanodisc lipids forming an annular shell around the TM region, which resembles the bilayer structure and mediates communication between the protein and lipid bilayer (Supplementary Movie 1). Notably, several lipid-binding sites in IP_3_R1 are located in intermolecular hydrophobic crevices, which are formed due to the domain-swapped architecture of the TMDs. As such, this allows one lipid to impart structural and functional influence to more than one subunit. The precise molecular identity and origin of protein-bound lipids is uncertain given that they could arise from either the native ER membrane or exogenous phosphatidylcholine added during protein solubilization in detergent and nanodisc formation.

In the IP_3_R1-LMNG map, lipid densities tightly associated with the TM domains were found at similar positions as in IP_3_R1-ND (Supplementary Fig. 5) suggesting strong lipid–IP_3_R1 interactions at these sites that may represent a specific lipid–protein architectural arrangement in the native lipid bilayer. Similar binding of lipid molecules in the hydrophobic crevices between adjacent subunits has also been shown in other homotetrameric ion channels. We found that the location of the L4-binding site between TM4-5, TM4, and TM3 helices is highly conserved among these ion channels (Supplementary Fig. 8).

Our observations led to the conclusions that the presence of protein-bound lipids stabilize the channel architecture, in particular the TM region, which shows higher local resolutions (2.4–3 Å) as compared to the CY domains (3-4.5 Å) (Supplementary Figs. 1e–g and 2d–f). In both the IP_3_R1-ND and IP_3_R1-LMNG structures, the conformation of the SF is wide enough to accommodate a fully hydrated Ca^2+^ ion, which is consistent with our previous cryo-EM structures of apo-IP_3_R1 determined in detergent^4,5^. These studies suggest that ligand-dependent activation on the channel involves conformational changes in the SF that could provide a plausible mechanism of Ca^2+^ recognition by the SF. Furthermore, we found that the pore-lining TM6 forms a continuous α-helical structure in both the lipid-based and detergent environments when the channel is visualized in the absence of activating ligands^4,5^. However, we have shown previously that a π-helix is observed in TM6 in the presence of activating ligands^5^. This observation raised the possibility that the α-to-π transition in TM6 underlies the gating in the IP_3_R1 channel. Notably, a π-helix has been also identified in pore-lining S6 of the superfamily of TRP channels^26,27,29^. It appears that TRP channels adopt the π-helical conformation of S6 quite differently upon their gating, with some channels utilizing an α-to-π transition, whereas some exhibit a π-to-α transition, and still others have the S6 π-helix in both closed and open states. Taken together, our results and aforementioned studies suggest that the structural mechanism for channel gating involving the α-to-π transition may not be generalized.

Furthermore, the two identified membrane-associated helices, MA1 and MA2, expand the membrane topology of IP_3_R1, and this finding reconciles a long-standing question on the correlation between TM domain predictions and cryo-EM structural observations. While there is no clear evidence for how these helices affect channel function, this region encompasses the determinants previously described to target IP_3_R to the ER^30,31^. Similar TMD features have begun to be described for RyR1^32,33^ and IP_3_R3^6^, albeit at lower resolutions. This suggests that the peripheral MA helices may be a conserved feature of the Ca^2+^ release channel family.

In summary, it is now largely appreciated that membrane lipids are integral components of ion channels, and by employing specific binding sites in the TM domains, lipids are likely to confer enhanced stability for proper channel gating. However, the exact molecular mechanism by which lipids exert their effects on IP_3_R channel function remains to be elucidated. It is conceivable that lipid binding to the protein may modulate the activation of the channel gate by providing a structural basis for integration and transmission of activating signals from primary stimuli, such as channel-specific ligands. Several Ca^2+^ signaling neurodegenerative diseases (e.g., Alzheimer and Huntington diseases), cardiac or muscular diseases (e.g., Duchenne muscular dystrophy) have been linked to abnormal Ca^2+^ signaling, as well as to altered membrane lipid composition, organization in lipid domains, and/or biophysical properties of membranes (Fig. 5). Given that IP_3_R channels play a key role in the control of cellular Ca^2+^ signaling, lipids might represent a missing link in human diseases, where initial perturbation of IP_3_R-lipid interactions resulting in altered regulation and gating of IP_3_R channels could lead to perturbations in cytosolic Ca^2+^ levels.

## Methods

### IP_3_R1 purification and reconstitution into nanodiscs

Purification steps were performed as previously described^4,8^ with the following modifications. Solubilization of native IP_3_R1 from rat cerebellum was carried out in 2 mM Lauryl Maltose Neopentyl Glycol (LMNG, Anatrace) and 0.1% (w/v) l-α-phosphatidylcholine (PC, Sigma) for 2 h at 4 °C. CnBr-sepharose beads (GE Healthcare) were coupled to purified monoclonal antibodies raised against the T433 epitope^34^. The hybridoma producing high-affinity monoclonal antibody against IP_3_R1 was raised in mice using synthetic peptide T433. The hybridoma was grown in serum-free media supplemented with 5% ultra-low IgG from fetal bovine serum (ThermoFisher Scientific). Antibodies were purified from culture supernatants by affinity chromatography using a fast flow protein G column (GE Healthcare) according to the manufacturer’s instructions. Immunoaffinity purification of IP_3_R1-LMNG was performed in 50 mM Tris-HCl (pH 7.4), 150 mM NaCl, 1 mM DTT, 1 mM EDTA, 0.02 mM LMNG and eluted with 500 μM of T433 peptide.

To reconstitute IP_3_R1s in lipid-nanodiscs, LMNG/PC-solubilized channels bound to the immunoaffinity resin were washed with ≥20 column volumes of 50 mM Tris-HCl (pH 7.4), 150 mM NaCl, 1 mM DTT, 1 mM EDTA, 0.02 mM LMNG. 1-palmitoyl-2-oleoyl-glycero-3-phosphocholine (POPC, Avanti Polar Lipids) was dried in a speedVac vacuum (1 h) and then resuspended (10 mg/ml) via probe sonication in 3% (w/v) n-dodecyl-β-d-maltoside (DDM, Avanti Polar Lipids, Inc), 50 mM Tris-HCl (pH 7.4), 150 mM NaCl, 1 mM DTT, 1 mM EDTA. Solubilized lipid was added to the column for a final concentration of 1.2 mM POPC and incubated for 30 min followed by the addition of 0.6 mg/ml MSP1E3D1 (Cube Biotech) dissolved in 50 mM Tris-HCl (pH 7.4), 150 mM NaCl, 1 mM DTT, 1 mM EDTA and 30 min incubation. To remove the detergents and allow for lipid-nanodisc reconstitution, activated Bio-Beads-SM2 (BioRad) were added to the immunoaffinity matrix at a final concentration of 0.25 mg/ml and incubated 16 h at 4 °C with the constant gentle rocking of the matrix. The matrix was washed with ≥20 column volumes of 50 mM Tris-HCl (pH 7.4), 150 mM NaCl, 1 mM DTT, 1 mM EDTA and IP_3_R1-ND was eluted with 500 μM of T433 peptide. The protein purification was assessed by 10% SDS PAGE (Supplementary Fig. 1a) with peak fractions concentrated to ~2 mg/ml using the 100 kDa Amicon centricon (Millipore) prior to immediate vitrification for cryo-EM imaging.

### Cryo-EM sample preparation

The purified IP_3_R1-ND (2 mg/ml) and IP_3_R1-LMNG (0.5 mg/ml) were incubated with 1 mM EGTA (apo-state) for 30 min on ice. The samples were vitrified by plunge-freezing in liquid ethane using a FEI Vitrobot Mark IV (ThermoFisher Scientific). 3.0 μl of IP_3_R1-ND or IP_3_R1-LMNG sample was applied onto a glow-discharged Quantifoil copper R 2/1 grid (Quantifoil Micro Tools, GmbH) containing a thin continuous carbon film, and blotted for 1–2 s at 100% humidity and 4 °C^5^.

### Cryo-EM data acquisition

Cryo-EM data collection was performed on a Titan Krios G3 microscope (ThermoFisher Scientific) operated at 300 kV and aligned for parallel illumination. BioQuantum energy filter (Gatan) was operated with an energy width of 20 eV. Images for both data sets were acquired using the EPU software (ThermoFisher Scientific) at a nominal TEM magnification of ×130,000 and recorded on a K2 Summit direct electron detector (Gatan) operated in super-resolution counting mode with a calibrated physical pixel size of 1.07 Å. The dose rate used on the detector was 8 electrons/Å^2^/s (for IP_3_R1-ND) and 6 electrons/Å^2^/s (for IP_3_R1-LMNG), respectively. The total exposure time of 7 s was fractionated into 35 subframes, each with 0.2 s exposure time, resulting in a total accumulated dose of 56 and 42 electrons/Å^2^ at the specimen plane, respectively. The images were acquired at a defocus range of 0.8–3.5 μm (Table 1). A total of 22,000 (IP_3_R1-ND) and 19,105 (IP_3_R1-LMNG) dose-fractionated movie stacks were collected as described above.

### Data processing

For both IP_3_R1-ND and IP_3_R1-LMNG data sets, movie stacks were motion-corrected with dose weighting and binned 2 × 2 by Fourier cropping resulting in a pixel size of 1.07 Å using MotionCor2^35^. The motion-corrected micrographs were evaluated by “*e2evalimage.py*” in EMAN2^36^, 18,547 micrographs (IP_3_R1-ND) and 17,280 micrographs (IP_3_R1-LMNG) were selected for subsequent processing using cryoSPARC^37^ and RELION3^38^.

For IP_3_R1-ND data, the motion-corrected micrographs were imported to cryoSPARC. The contrast transfer function (CTF) was determined using CTFFIND4^39^. ~1000 particles were selected manually for 2D classification and the selected 2D class-averages were used for template-based autopicking. A total of 4,801,610 initial particle picks were subjected to 2D classification, identifying 1,495,402 particles that were subjected to further processing in cryoSPARC. Our previously published map (EMDB-9246) was low pass filtered to 60 Å resolution and used as an initial model for Heterogeneous 3D Refinement with C1 symmetry. 573,723 particles from the best 3D classes were subjected to Homogeneous 3D refinement with a soft mask using C4 symmetry. The final round of refinement and post-processing were carried out in RELION3.

For IP_3_R1-LMNG data, 1,407,714 particles were extracted using the NeuralNet autopicking procedure implemented in EMAN2^40^ and were imported to RELION3 for the following image processing. The CTF parameters were determined using GCTF^41^. We then followed up with 2 rounds of 2D classification that yielded 1,011,190 particles (Supplementary Fig. 2b). These particles were subjected to 3D classification performed without imposing any symmetry. Our published map (EMDB-9246), low-pass filtered to 60 Å resolution, was used as an initial model. Based on 3D classification results, the best 303,481 particles were selected and subjected to further 3D refinement by applying C4 symmetry and a soft mask.

For both maps, the post-processing step was performed in RELION3, a soft mask was calculated and applied to the two half-maps before the Fourier shell correlation (FSC) was calculated. *B*-factors were estimated and applied to the map sharpening step (Supplementary Figs. 1d and 2c). The resolutions for the final 3D reconstructions using the standard 3D refinement approach were 3.30 Å for IP_3_R1-ND and 2.96 Å for IP_3_R1-LMNG based on the gold standard criteria^42,43^. Local resolution variations were estimated using ResMap^44^ (Supplementary Figs. 1g and 2f).

### Model building and refinement

The same strategy was utilized for building 3D models for both the IP_3_R1-ND and IP_3_R1-LMNG cryo-EM density maps. Initial models were generated from our previously published cryo-EM structure of apo-IP_3_R1 (PDB ID: 6MU2) using rigid body fitting tool in UCSF Chimera^45^. “phenix.resolve_cryo_em” density modification^46^ was performed on the half-maps resulting in improved resolvability in the density maps allowing for further model optimization. After rigid-body fitting, initial flexible fitting was performed in COOT^47^ by manually adjusting the entire protein-peptide chain of a single IP_3_R1 subunit. After the coarse manual fit, real-space refinement was performed with “phenix.real_space_refine”^48^ and subsequently adjusted again manually in Coot. This process was repeated in order to maximize fit to density, minimize Ramachandran angle outliers and eliminate steric clashes. Further model validation was carried out using EMRinger^49^ and MolProbity^50^ in PHENIX. The full tetrameric model was completed by calculating map symmetry in UCSF Chimera and applying it to the model. A final round of real-space refinement in Phenix and manual optimization in Coot was performed on the entire tetramer before modeling the lipids.

Non-IP_3_R1 protein densities visualized in the cryo-EM maps were isolated by subtracting the protein model from the corresponding map using the “zone” tool in UCSF Chimera^45^. Nanodisc lipids and MSP appear to form a toroidal layer of densities in the IP_3_R1-ND reconstruction. Lipid-like densities with a characteristic head-and-two-tails shape were found to be associated with the TM domains of IP_3_R1-ND and IP_3_R1-LMNG structures. These densities were putatively modeled as phosphatidylcholine (PC) lipids (https://pubchem.ncbi.nlm.nih.gov), which are a major component of ER membranes (>50%)^10^ and were also added during the protein solubilization. The PC models were obtained from PubChem (https://pubchem.ncbi.nlm.nih.gov) and fit into the corresponding cryo-EM densities using Coot. The final full refinement of the entire IP_3_R1-lipid model was performed in Phenix using “phenix.real_space_refine”. The overall PDB model validation statistics are presented in Table 1.

The final IP_3_R1-ND model includes the following residues: 6–323, 354–532, 536–674, 693–898, 960–1008, 1025–1045, 1055–1130, 1170–1538, 1598–1690, 1724–1745, 1786–1882,1955–2134, 2146–2476, and 2524–2739. The IP_3_R1-LMNG model consists of residues: 6–323, 354–532, 536–674, 693–898, 960–1008, 1025–1045, 1055–1130, 1170–1538, 1598–1690, 1724–1745, 1786–1882, 1955–2134, 2146–2476, and 2524–2741 (Supplementary Table 1). The residue numbering includes the first methionine according to the primary sequence with the GenInfo Identifier (GI) code 17380349. The full model vs. map FSC plots was calculated using MTRIAGE^51^ validation tool in PHENIX (Supplementary Figs. 1h and 2g). Noteworthy, the map regions with lower-than-average resolution (Supplementary Figs. 1g and 2f) were only modeled with mainchain atoms (e.g., ARM2 domain and MA helices) or not modeled at all (Supplementary Table 1). This resulted in a shift of the fall-off to a lower resolution in the FSC between the final refined model and cryo-EM map (Supplementary Figs. 1h and 2g). While map-vs-model FSC for the extracted TM domains, calculated using EMAN2^40^, indicated that the resolution based on the FSC = 0.5 criteria is consistent with the gold-standard resolution at the FSC 0.143 cut-off (Supplementary Figs. 1i and 2h).

Map-model visualization was performed in Coot and UCSF Chimera. Interfaces described in the paper were identified with PDBSum^52^, PDBePisa^53^, and HOLE^54^. The figures and movie were produced using UCSF Chimera and VMD 1.9.4.^55^.

### Statistics and reproducibility

No statistical method was used to predetermine the sample size, and the experiments were not randomized. Each cryo-EM data set was collected from one grid. Individual images with bad ice were excluded from the data set by visual inspection. Data collection, processing, and refinement statistics were summarized in Table 1.

### Reporting summary

Further information on research design is available in the Nature Research Reporting Summary linked to this article.

## Supplementary information

Supplementary Information

Description of Additional Supplementary Files

Supplementary Video 1

Supplementary Video 2

Reporting Summary

## Data Availability

The cryo-EM density maps of IP_3_R1 have been deposited to the Electron Microscopy Data Bank under accession numbers EMD-23337 (IP_3_R1-ND) and EMD-23338 (IP_3_R1-LMNG). Atomic coordinates have been deposited to the Protein Data Bank (PDB) with accession codes 7LHE (IP_3_R1-ND) and 7LHF (IP_3_R1-LMNG).
