# Supplementary material for: Cryo-EM structure of type 1 IP3R channel in a lipid bilayer
Source: Commun Biol. 2021 May 25;4:625. doi: 10.1038/s42003-021-02156-4 (PMC8149723; doi:10.1038/s42003-021-02156-4)
Supplement: Supplementary file 1 — Supplementary Information [file 42003_2021_2156_MOESM1_ESM.pdf]

SUPPLEMENTARY FIGURES

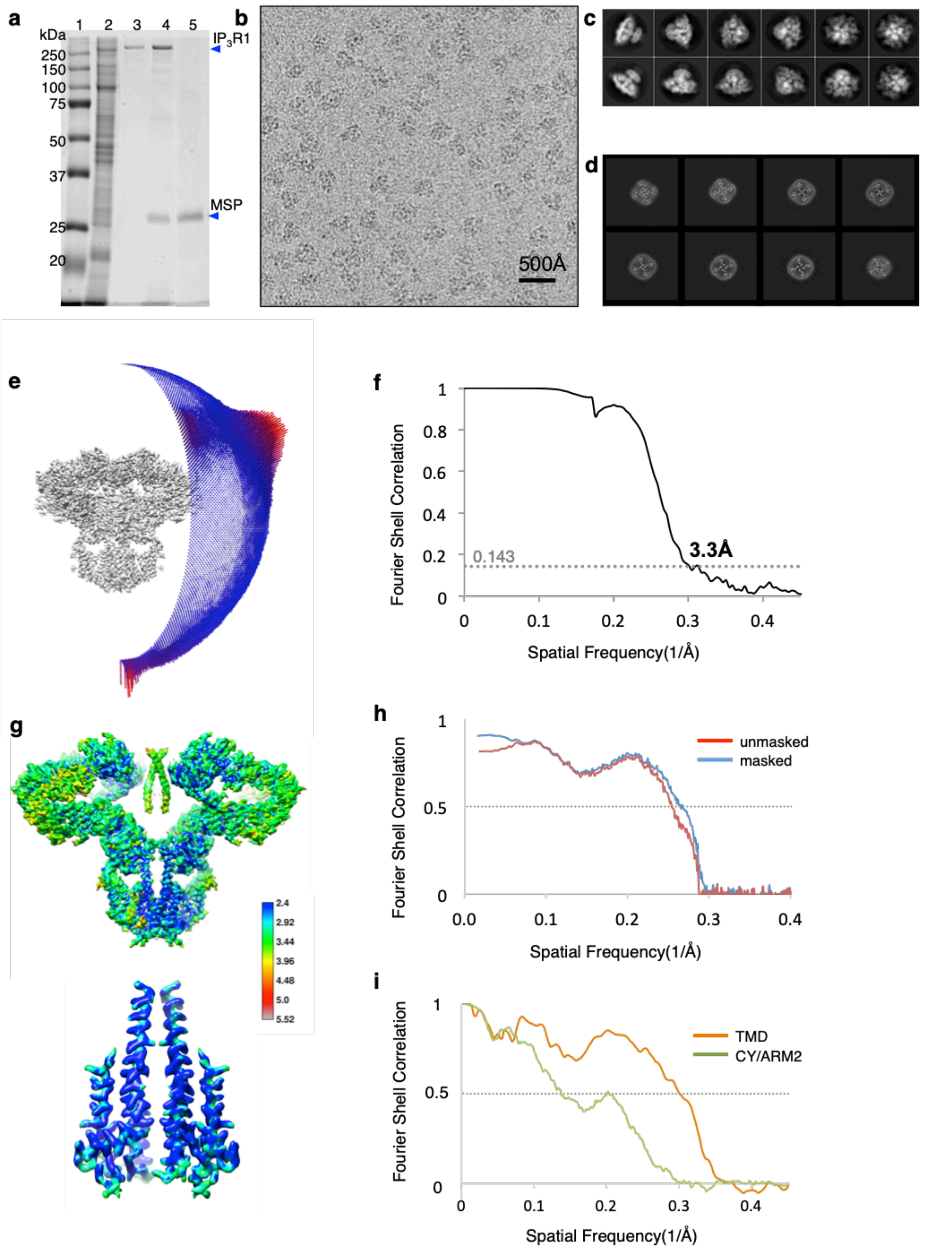

Supplementary Figure 1. The cryo-EM structure of ligand-free IP$_3$R1 channel in nanodisc. a, SDS-PAGE gel: lane 1- molecular weight standards; 2 - rat cerebellar membranes; 3 - purified IP$_3$R1 in LMNG; 4 - purified IP$_3$R1 in nanodisc; 5 - MSP1E3D1/nanodiscs. **b**, Representative raw micrograph of ice-embedded IP$_3$R1-ND. **c**, Representative 2D class averages. **d**, Slices through the unsharpened density map along the 4-fold axis at TM domain. **e**, Angular orientation distribution of all particles used in the final 3D reconstruction. **f**, The gold-standard FSC curve for the final reconstruction indicates an overall resolution of 3.30 Å at the 0.143 FSC cut-off. **g**, Density map of two opposing subunits of IP$_3$R1-ND is color-coded based on ResMap, bottom panel: solvent-accessible pathway along the pore. **h**, The FSC curve between the cryo-EM map and the entire model of IP$_3$R1-ND (Methods); **i**, Map vs. model FSC curves calculated for the TMD (residues 2273-2296, 2350-2595, orange) and the cytosolic ARM2 domain (residues 1025-1538, green) indicate 3.31 Å and 7.2 Å resolution, respectively, at the 0.5 FSC cut-off.

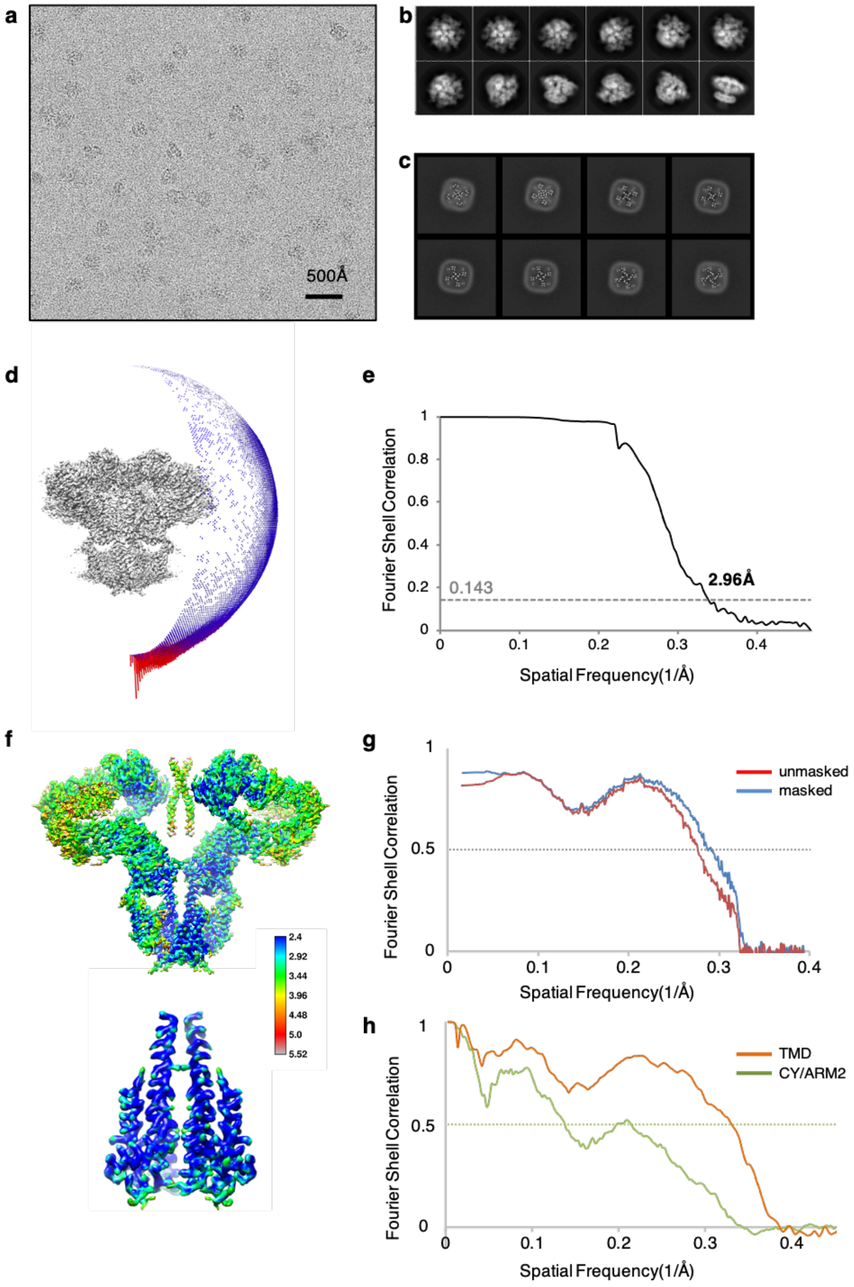

Supplementary Figure 2. The cryo-EM structure of ligand-free IP$_3$R1 channel with LMNG. a, Representative raw micrograph of ice-embedded IP$_3$R1-LMNG. **b**, Representative 2D class averages. **c**, Slices through the unsharpened density map along the 4-fold axis at TM domain. **d**, Angular orientation distribution of all particles used in the final 3D reconstruction. **e**, The gold-standard FSC curve for the final cryo-EM 3D reconstruction indicates an overall resolution of 2.96 Å at the 0.143 FSC cut-off. **f**, Density map of IP$_3$R1-LMNG is color-coded based on ResMap (Methods), bottom panel: solvent-accessible pathway along the pore. **g**, The FSC curve between the cryo-EM map and the entire model of IP$_3$R1-LMNG (Methods); **h**, Map vs. model FSC curves calculated for the TMD (residues 2273-2296, 2350-2595, orange) and the cytosolic ARM2 domain (residues 1025-1538, green) indicate 3.03 Å and 7.3 Å resolution, respectively, at the 0.5 FSC cut-off.

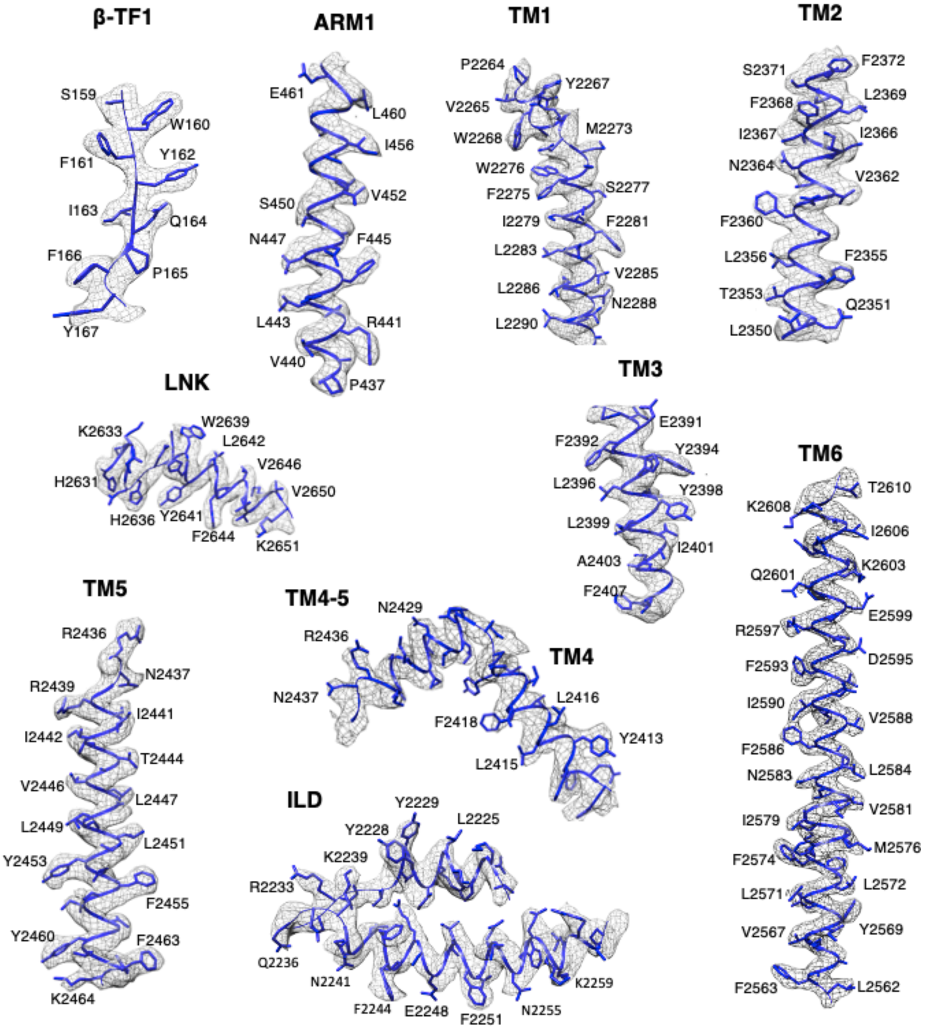

Supplementary Figure 3. Representative cryo-EM densities of IP$_3$R1 in nanodisc for selected regions are overlaid with corresponding models.

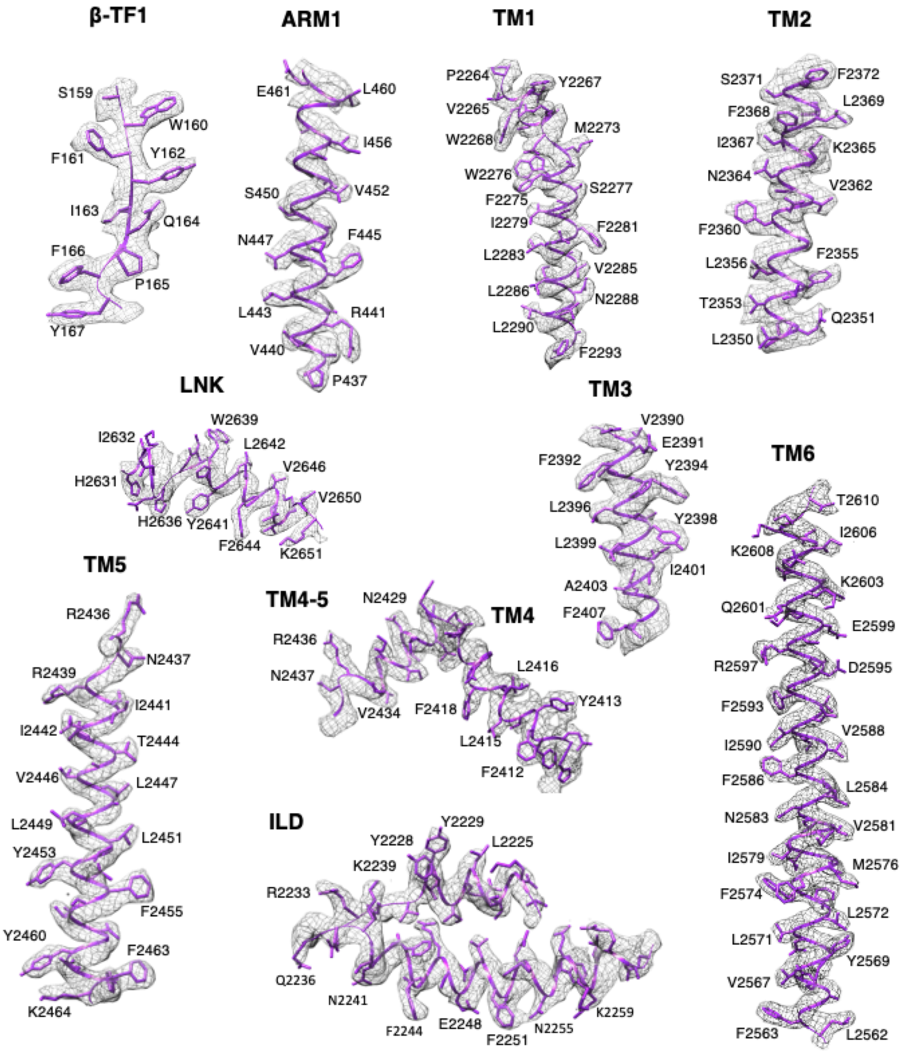

Supplementary Figure 4. Representative cryo-EM densities of IP$_3$R1 in LMNG for selected regions are overlaid with corresponding models.

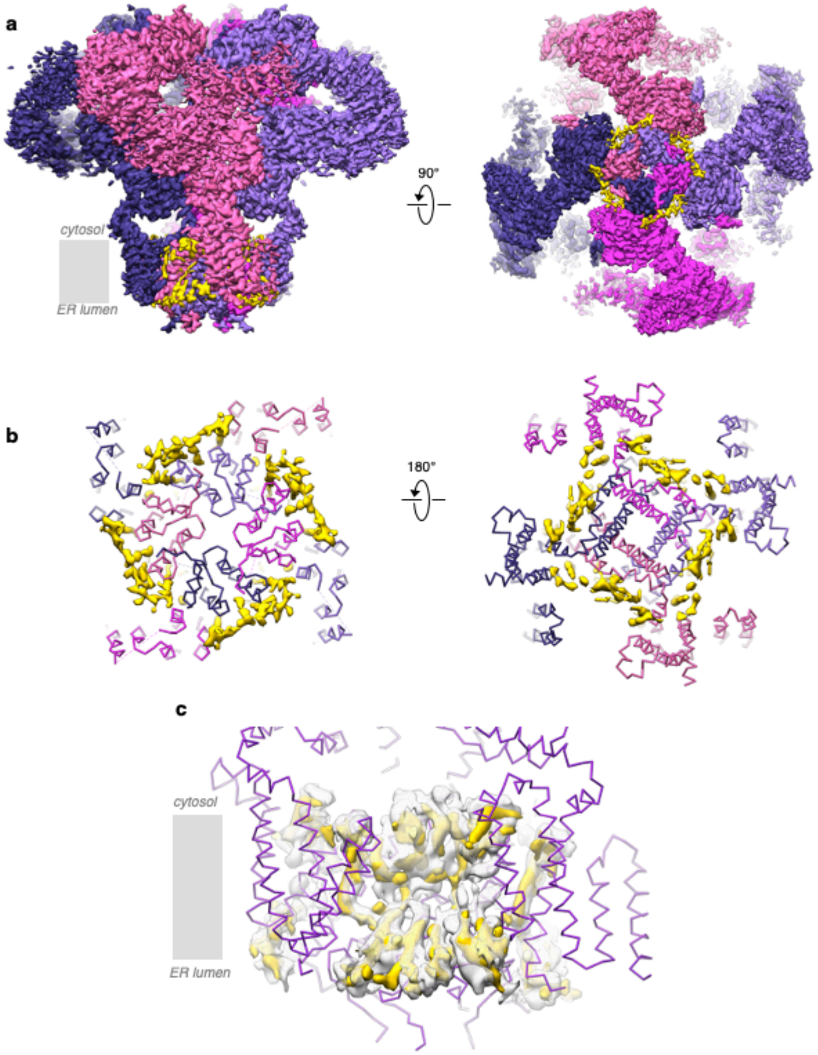

Supplementary Figure 5. The cryo-EM structure of the IP_3R1 channel in LMNG. a, The cryo-EM density map of the IP_3R1-LMNG is viewed along the membrane plane (left) and from the lumen along the four-fold axis (right). Individual subunits are color-coded. Densities corresponding to lipids are colored yellow. **b,** Distribution of lipids (yellow) in inter- and intra-subunit crevices viewed along the four-fold axis from the lumen (left) and cytosol (right). **c,** Shown are lipids densities in IP_3R1-LMNG (yellow) and IP_3R1-ND (grey) displayed at 3σ.

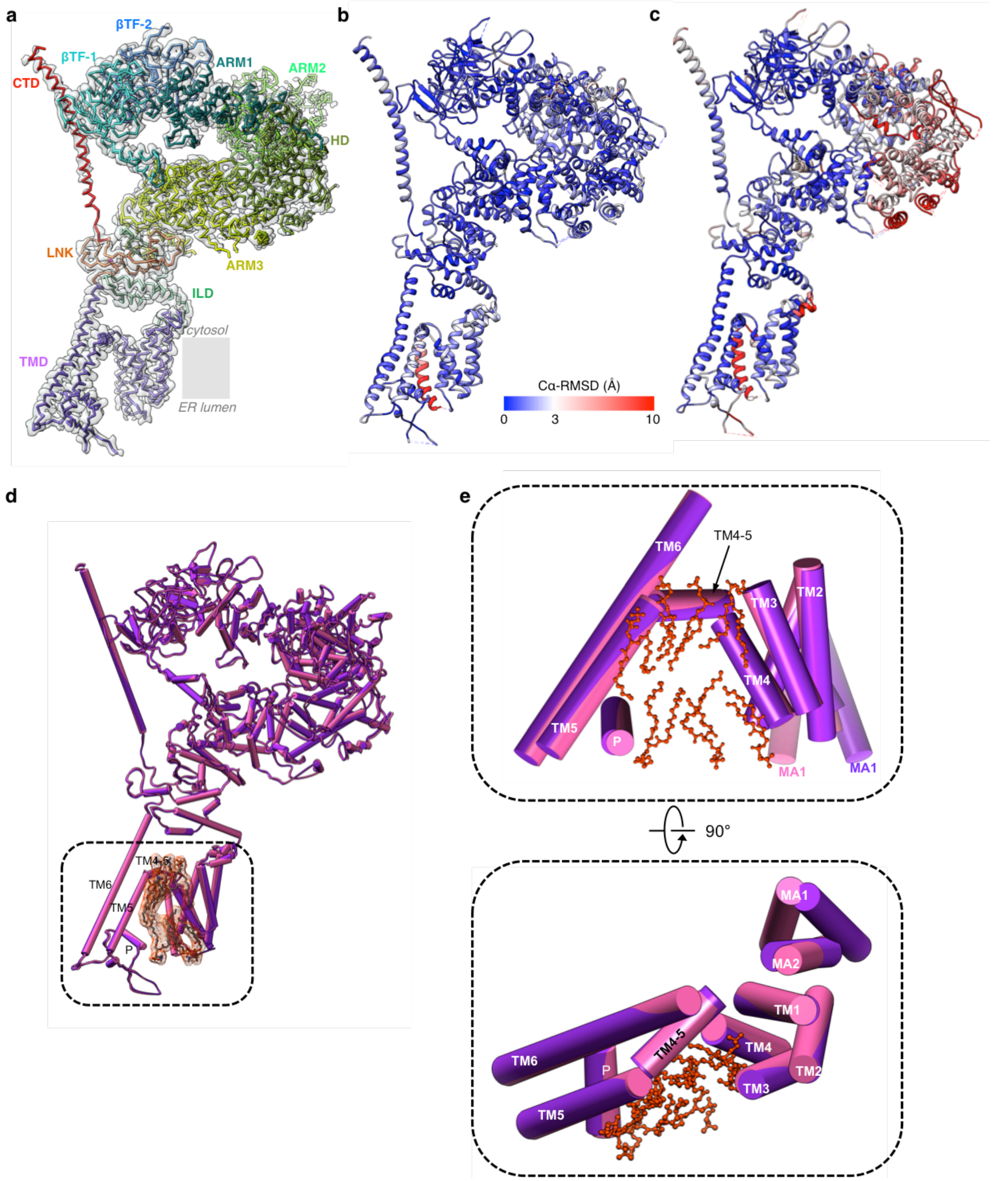

Supplementary Figure 6. Comparison of IP$_3$R1 structures in nanodisc and detergent. a, The cryo-EM density map for one subunit of IP$_3$R1-ND is overlaid with the model. The domains are color-coded and labeled[4]. **b**, An individual subunit of IP$_3$R1-ND depicted as a ribbon model and color-coded based on the Cα RMS deviations calculated between (**b**) IP$_3$R1-ND and IP$_3$R1-LMNG structures and between (**c**) IP$_3$R1-ND and IP$_3$R1-CHAPS (PDB:6MU2) structures. **d**, Individual subunits from IP$_3$R1-ND (pink) and IP$_3$R1-LMNG (purple) structures are superimposed; helices are depicted as cylinders and viewed along the membrane plane. Lipid molecules are orange and overlapped with corresponding surface models. **e**, Zoomed in region of the TMD indicated by the black dashed line in '**d**', viewed along the membrane plane (top panel) and viewed from the cytosol along the four-fold axis (bottom panel).

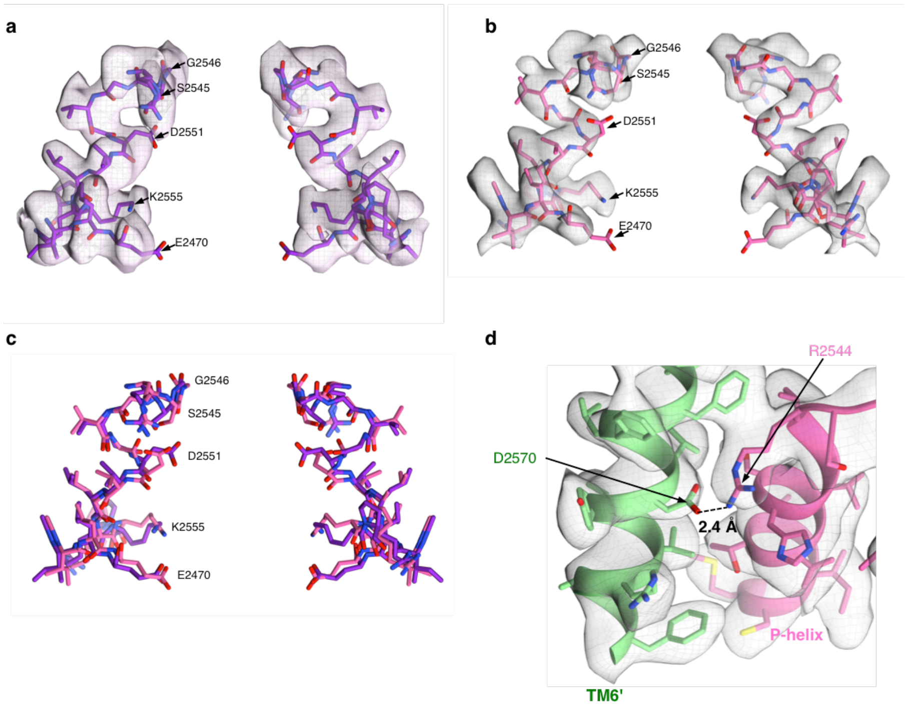

Supplementary Figure 7. Structure of the P-helix and SF in IP$_3$R1 channel. a, SF of IP$_3$R1-LMNG and (**b**) IP$_3$R1-ND are viewed along the membrane plane as depicted in Fig. 2. Cryo-EM density maps are shown in grey. **c**, SF of two opposing subunits from IP$_3$R1-LMNG (purple) and IP$_3$R1-ND (pink) are overlayed. **d**, Putative intermolecular interactions between the P-helix and the TM6 helix of the adjacent subunit. Cryo-EM density map is shown in grey and the model is displayed as a ribbon and colored by subunit.

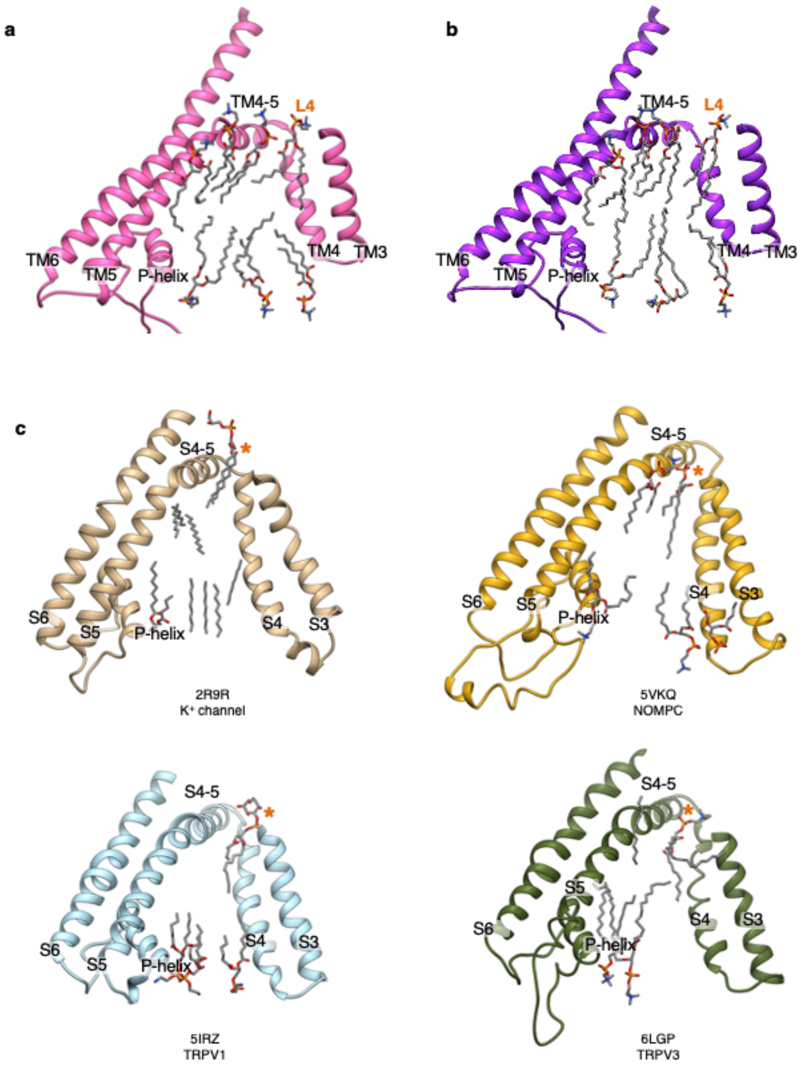

Supplementary Figure 8. Comparison of locations of lipids bound to TM domains in (**a**) IP_3R1-ND and (**b**) IP_3R1-LMNG with those in (**c**) other tetrameric cation channels: Kv1.2-2.1, NOMPC, TRPV1 and TRPV3 (PDB accessions: 2R9R, 5VKQ, 5IRZ and 6LGP respectively). Lipids are color-coded by elements. L4 in IP_3R1-ND/IP_3R1-LMNG and corresponding lipid molecules identified in other tetrameric channels at near identical locations are labeled with an orange star.

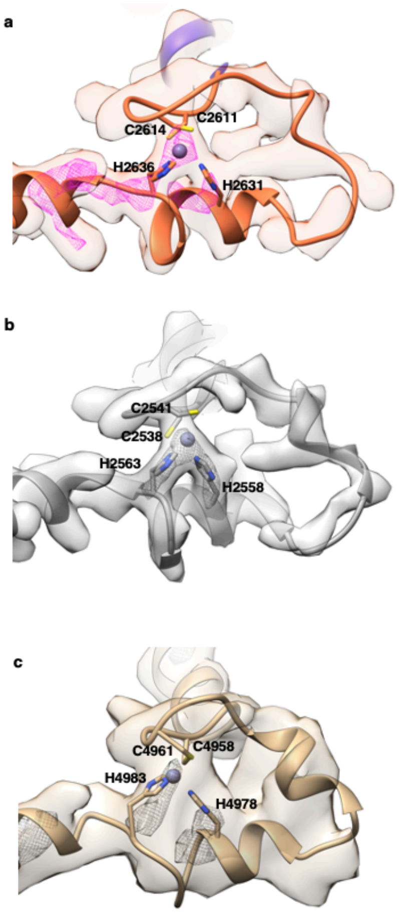

Supplementary Figure 9. Zn^{2+} binding site in calcium release channels. The Zn^{2+} finger domains in apo-IP_3R1-ND (**a**), apo-IP_3R3 (**b**, EMDB-7978) and apo-RyR1 (**c**, EMDB-8387). The mesh densities are displayed at 24σ for IP_3R1 and 12σ for IP_3R3 and RyR1. Residues that contribute to Zn^{2+} binding are shown.

IP_3R1-ND<br>EMD-23337<br>PDB 7LHE		IP_3R1-LMNG<br>EMD-23338<br>PDB 7LHF	
All-atom model	Backbone only model	All-atom model	Backbone only model
6-323*		6-323*	
354-532		354-532	
536-674		536-674	
693-898		693-898	
960-1008		960-1008	
1025-1045		1025-1045	
1055-1130		1055-1130	
1170-1462		1170-1361	
	1463-1538		1362-1538
1598-1690		1598-1690	
1724-1745		1724-1745	
1786-1882		1786-1882	
1955-2134		1955-2134	
2146-2300		2146-2300	
	2301-2346		2301-2346
2347-2476		2347-2476	
2524-2739		2524-2741	

*Residues are numbered according to the primary sequence from GI 17380349.

Supplementary Table 1. Summary of modeling.
