# Supplementary material for: Cryo-EM structure of type 1 IP3R channel in a lipid bilayer
Source: Commun Biol. 2021 May 25;4:625. doi: 10.1038/s42003-021-02156-4 (PMC8149723; doi:10.1038/s42003-021-02156-4)
Supplement: Supplementary file 2 — Description of Additional Supplementary Files [file 42003_2021_2156_MOESM2_ESM.pdf]

Description of Additional Supplementary Files

File name: Supplementary Movie 1

Description: Cryo-EM structure of the IP3R1 in nanodisc.

File name: Supplementary Movie 2

Description: Comparison of MA helices in IP3R1-ND and IP3R1-LMNG.
